# Predicting heterosis *via* genetic distance and the number of SNPs in selected segments of chromosomes in maize

**DOI:** 10.3389/fpls.2023.1111961

**Published:** 2023-02-17

**Authors:** Fuyan Jiang, XingFu Yin, Zi Wei Li, Ruijia Guo, Jing Wang, Jun Fan, Yudong Zhang, Manjit S. Kang, Xingming Fan

**Affiliations:** ^1^ College of Agronomy and Biotechnology, Yunnan Agricultural University, Kunming, Yunnan, China; ^2^ Institute of Food Crops, Yunnan Academy of Agricultural Sciences, Kunming, Yunnan, China; ^3^ Yunnan Dehong Dai and Jingpo Nationality Institute of Agricultural Sciences, Mangshi, Yunnan, China; ^4^ Department of Plant Pathology, Kansas State University, Manhattan, KS, United States

**Keywords:** maize, grain yield, heterosis prediction, SNP, genetic distance

## Abstract

A reliable method is needed for predicting heterosis to help maize (*Zea mays* L.) breeders develop new hybrids more efficiently. The objectives of this study were to 1) investigate if the numbers of selected PEUS SNPs (the SNP in the Promoters (1 kb upstream of the start codon), Exons, Untranslated region (UTR), and Stop codons) could be used for predicting MPH or BPH of GY; 2) if the number of PEUS SNPs is a better predictor of MPH and/or BPH of GY than genetic distance (GD). A line × tester experiment was conducted with 19 elite maize inbreds from three heterotic groups, which were crossed with five testers. The multi-location trial data on GY were recorded. Whole-genome resequencing of the 24 inbreds was carried out. After filtration, a total of 58,986,791 SNPs were called with high confidence. Selected SNPs in the promoters, exons, untranslated region (UTRs), and stop codons (PEUS SNPs) were counted, and the GD was calculated. The correlation between heterozygous PEUS SNPs/GD and mean MPH, BPH of GY revealed that 1) both the number of heterozygous PEUS SNP and the GD were highly correlated to both MPH_GY and BPH_GY at p<0.01 with correlation coefficients for the number of heterozygous PEUS SNP being higher than that for GD; 2) the mean number of heterozygous PEUS SNPs was also highly correlated with mean BPH_GY or mean MPH_GY (p<0.05) in the 95 crosses grouped by either male or female parents, implying that inbreds can be selected before making the actual crosses in the field. We concluded that the number of heterozygous PEUS SNPs would be a better predictor of MPH_GY and BPH_GY than GD. Hence, maize breeders could use heterozygous PEUS SNPs to select inbreds with high heterosis potential before actually making the crosses, thus improving the breeding efficiency.

## Introduction

A reliable method for predicting heterosis will greatly facilitate maize breeders to develop new hybrids with greater efficiency; researchers put forth continuous efforts to explore this. Maize is one of the earliest crops in which heterosis was discovered. In 1907, Shull put forward the concept of heterosis for the first time, laying the theoretical foundation of modern maize breeding. Since then, many investigations have been conducted, and thoughtful articles have been written to understand the mechanisms of heterosis. Kaeppler (2012) suggested that sequence diversity was necessary but not sufficient to produce heterotic phenotypes and that there were many diverse molecular mechanisms underlying heterosis. He further indicated that a combination of various mechanisms produced heterosis in complex traits. Rehman et al. (2021) reviewed various studies on heterosis and pointed out that increased knowledge across fields of science, such as genetics, epigenetics, genomics, proteomics, and metabolomics, provided new insights into understanding the expression of hybrid vigor or heterosis.

Mid-parent heterosis (MPH) and better-parent heterosis (BPH) are widely used for selecting hybrids in maize breeding programs (Fan et al., 2008a; Fan et al., 2008b; Fan et al., 2009; Kumar et al., 2019). MPH_GY is most frequently measured for selecting high-yielding maize hybrids (Garcia et al., 2009; Zhang et al., 2016). The line × tester method is widely used for computing general combining ability and specific combining ability and for identifying new high-yielding hybrids (Fan et al., 2016; Jiang et al., 2020). Undoubtedly, the information on BPH and MPH for grain yield (GY) in maize is highly useful in determining which maize lines should be selected to improve the local lines and which parental lines should be crossed for developing high-yielding hybrids (Fan et al., 2008b).

Molecular markers and genetic distance (GD) are extensively used in maize heterotic group classification, GY, and heterosis prediction in maize (Schrag et al., 2006; Schrag et al., 2010; Singh et al., 2018; Nyaga et al., 2020; Sang et al., 2022). Schrag et al. (2006) tried to predict the GY of single-cross hybrids *via* amplified fragment length polymorphism (AFLP) associated with quantitative trait loci (QTL). They suggested that it was advantageous for predicting single-cross hybrids to enhance a general combining ability-based model. Schrag et al. (2010) compared the prediction efficiency of AFLPs and simple sequence repeat (SSR) markers for selecting hybrids. No clear-cut results were obtained with AFLPs and SSRs, and they concluded that joint analyses of hybrids and parental inbred lines might have a higher potential for predicting the performance of untested hybrids. Singh et al. (2018) investigated the relationship between genetic distance and BPH using SSRs and SNPs. They found that the correlation between GD and BPH for GY was positive and significant but small (r = 0.33 with SSR, p< 0.01; r = 0.35 with SNPs, p< 0.01). Nyaga et al. (2020) investigated if molecular markers could predict the heterosis of maize hybrids from inbred lines. A low and even negative correlation was observed between the parental lines’ SNPs and heterosis. Sang et al. (2022) demonstrated that the GD between the parental lines within the heterotic groups was significantly correlated with hybrid GY and MPH of GY.

Previous studies have indicated that molecular markers alone or in conjunction with other measures could be employed for predicting the GY and/or MPH_GY or BPH_GY (Singh et al., 2018; Nyaga et al., 2020; Sang et al., 2022) and various types of markers used seemed to improve the prediction accuracy. However, the prediction of heterosis *via* molecular markers was not conclusive. Gavora et al. (1996) reported that SNPs could reduce the functioning of genes by altering the activity or production of enzymes, or by reducing the efficiency of transcription factor binding. Loss-of-function could result from SNPs producing nonsense alleles or altering splice junctions, loss of transcript due to the absence of a sequence or by epigenetic silencing. In maize, high-density SNP markers are well distributed in the QTL or genes and even exons and promoters. The PEUS SNPs are selected from the promoters, exons, untranslated regions (UTRs), and stop codons. The PEUS SNPs are the key SNPs that may be related to the gene expression, which, in turn, determines the traits or phenotype of an individual (Savas et al., 2006; Kim et al., 2008; Vage and Lingaas, 2008; Pan et al., 2021). The questions that need to be asked are: 1) are the heterozygous PEUS SNPs and GD calculated with all genetic markers in two inbreds of maize related to the MPH and/or BPH of a hybrid produced from the two inbreds? 2) can the SNP information from an inbred be used for inbred selection to develop crosses with high MPH or BPH of GY? To address these questions, a line × tester experiment was carried out with 19 elite maize lines crossed with five testers (i.e., four RILs and their recurrent parent (Q11)). The specific goals of this study were 1) to investigate whether the numbers of selected heterozygous PEUS SNPs, as GD does, can predict the MPH or BPH of GY; 2) whether the heterozygous PEUS SNP information better predicts the MPH and/or BPH for GY than GD does.

## Materials and methods

### Plant materials

Information on pedigree, heterotic group, and ecological adaptation of the 19 inbred lines (female) and five testers (male) used in this experiment is given in **Table 1**. The 19 inbred lines were selected on the basis of previously determined GY and genetic diversity. Testers consisted of four RILs, developed by Li et al. (2018), and Q11, a widely used elite maize line in China. In the 2017 winter season, the five testers were crossed with the 19 inbred lines (female parents) in a line × tester design at Dehong, Yunnan, China, to generate 95 crosses.

**Table 1:** The pedigree of 19 lines and five testers and their ecological adaptation.

Line code†	Line name	Pedigree	Heterotic group	Adaptation environment
L1	CML312	S89500-F2-2-2-1-1-B	nonReid	Tropical
L2	CML373	P43SR-4-1-1-2-1-B-8-1-B	nonReid	Tropical
L3	CML395	90323B-1-B-1-B	nonReid	Tropical
L4	YML32	Selected from Suwan1	Suwan1	Tropical
L5	YML226	(CML226/(CATETO DC1276/7619))F2-25-1-B-1	nonReid	Tropical
L6	TML139	Selected from Suwan1	Suwan1	Tropical
L7	TRL2	Derived from US hybrid	nonReid	Subtropical
L8	3760	Derived from South Africa hybrid	nonReid	Subtropical
L9	Zheng58	Derived from Ye 478	Reid	Temperate
L10	Y1218	HuangZhaoShi×WeiChun	nonReid	Temperate
L11	Chang 7-2	V59× HuangZhaoShi	nonReid	Temperate
L12	Huang C	Yugoslavia O2/Huangxiao 162/Zi330/Mobai 1	nonReid	Temperate
L13	YML46	Selected from Suwan1	Suwan1	Tropical
L14	YML16	GLSIY01HGB-B-27-1-2-B	nonReid	Tropical
L15	CML171	Pool25QPM	nonReid	Tropical
L16	AN20	Derived from US hybrid	nonReid	Subtropical
L17	NK40-1	Derived from US hybrid	Reid	Tropical
L18	R-2-1-1	Derived from US hybrid	Reid	Temperate
L19	Shen137	Derived from US hybrid(6JK111)	nonReid	Temperate
RL1_1		Y32/Q11-BC1-1-1-1-1 with GZ204/IDP5	Reid	Temperate
RL1_2		Y32/Q11-BC1-1-1-1-2 with GZ204/IDP5	Reid	Temperate
RL2_1		Y32/Q11-BC2-1-1-1-1-1 with GZ204/IDP5	Reid	Temperate
RL2_2		Y32/Q11-BC2-1-1-1-1-2 without GZ204/IDP5	Reid	Temperate
Q11		Recurrent parent, without GZ204/IDP5	Reid	Temperate

†Lines L1 to L19 were used as female parents and RL1_1, RL1_2, RL2_1, RL2_2 and Q11 were used as testers (male parents).

### Field trials and data collection

Four recombinant inbred lines (RILs) were developed by crossing a Suwan1 inbred YML32 with a widely used in China Reid inbred line ‘Q11’ (Li et al., 2018). YML32 is a tropical inbred line selected from the Suwan1 population by the Institute of Food Crops, Yunnan Academy of Agricultural Sciences, China. It was used as the male parent for developing gray leaf spot (GLS)-resistant hybrid Yunrui 1 (Liu et al., 2016). The GLS-susceptible line Q11 was derived from HL1999, which had a high combining ability for GY and was widely used in maize breeding programs in southwest China. A line × tester experiment was conducted with five testers (i.e., the four RILs and their recurrent parent line Q11, used as ♂) and 19 elite maize inbreds (used as ♀).

The 95 crosses, along with their parents and one check (Yunrui62), were planted in the summer of 2018 (Experiment 2018) at three locations in Yunnan (China): Dehong (24°26 ‘N, 98°35 ‘E, elevation: 914 masl), Kunming (25°23 ‘N, 102°9 ‘E, elevation: 1970 masl) and Yanshan (23°60 ‘N, 104°4 ‘E, elevation: 1570 masl). A randomized complete block design with three replications was employed at each location. The experimental plot consisted of a single 3.5-m-long row, with a plot-to-plot spacing of 0.70 m and plant-to-plant spacing of 0.25 m within a row. Four seeds per hill were sown. Upon germination, thinning was done, and two seedlings per hill were maintained. The resulting plant density was approximately 60,000 plants ha^-1^. Trials were managed according to standard agronomical practices. The GY was determined at each location from 10 plants taken from the middle of each row and was expressed as tons ha^-1^.

### Statistical analysis

The following general linear model was used for the analysis of variance (ANOVA) for GY


$$Y_{ijkl}=\;\mu \;+\;\alpha_{l}+b{\left(a\right)}_{kl}+\upsilon_{ij}+\;{\left(\alpha \upsilon \right)}_{ijl}+e_{ijkl}$$

$$\upsilon_{ij}=\;l_{i}+\;t_{j}+\;lt_{ij}$$

where *Y_ijkl_* = observed value from each experimental unit; μ = population mean; *α_l_* = location effect; *b(a)_kl_* = replication effect within each location; *υ_ij_* = F_1_ hybrid effect = l*_i_* + t*_j_* + lt*_ij_* (where l*_i_* = *i*th line effect; t*_j_* = *j*th tester effect; lt*_ij_* = interaction effect between *i*th line and *j*th tester); (*αυ*)*_ijl_* = interaction effect between *ij*th F_1_ hybrid and *l*th location; and *e_ijkl_* = residual effect.

The locations were treated as a fixed effect. Since the lines and testers were not selected at random, significance for lines, testers, and lines × testers and their interactions with the locations was determined against the overall experimental error term. Combining ability analysis was conducted according to the method proposed by Fan et al. (2009). Mid-parent heterosis (MPH) was determined as follows:

MPH = [(F_1_ – MP)/MP] × 100

where F_1_ = Mean of hybrid, MP = Mid-parent value = (P1 + P2)/2; where P1 = Parent 1 and P2 = Parent 2.

BPH = [(F_1_ – BP)/BP] × 100

where F_1_ = Mean of hybrid, BP = Better-parent value.

Fisher’s least significant difference (LSD) was used to separate MPH and GY means of testcrosses. Data analysis was conducted *via* the SAS 9.1.3 software package (SAS Institute, 2005).

Identity by state (IBS) distance matrix was applied to explain the genetic kinship between individuals within a group calculated by PLINK v.107. software (Purcell et al., 2007). The genetic distance (GD) between two parents of each hybrid was calculated as GD = 1- IBS (Geng et al., 2021).

### SNP selection

Raw sequences with a 150-bp read length for the 24 parental lines were generated *via* the Illumina Novaseq platform (Illumina, San Diego, CA, USA). After quality checking and filtering, the high-quality paired-end reads were mapped to the B73 reference genome (Jiao et al., 2017) using Burrows-Wheeler Aligner software (Li and Durbin, 2009), with the parameters of ‘mem -t 4 -k 32 -M’. SNPs were then called using the Genome Analysis Toolkit software (McKenna et al., 2010) based on the B73 reference genome (Jiao et al., 2017). Finally, high-quality SNPs were filtered with the following parameters: depth for each individual ≥ 3, base quality ≥30, genotype quality for each individual ≥ 5, with a missing rate of ≤ 0.2. The identified SNPs were further annotated using the ANNOVAR software tool (v2013-05-20) (Wang et al., 2010).

Based on the ANNOVAR annotation, the SNPs in the promoters (1 kb upstream of the start codon), exons, untranslated regions, and stop codons were selected for analysis. These SNPs were designated as PEUS SNPs. If PEUS SNPs were the same at a point or locus, they were considered homozygous SNPs; if the PEUS SNPs were different, they were considered heterozygous SNPs. Then homozygous SNPs and heterozygous SNPs were counted in the 95 crosses and mean homozygous PEUS SNPs and mean heterozygous PEUS SNPs were calculated for the five testers and 19 lines.

## Results and discussion

### ANOVA for GY, ED, EL, RE, KR, and HKW

Analysis of variance (ANOVA) results for GY is given in **Table 2**. The mean squares for lines, testers, lines × testers, and locations for GY were statistically significant. These results implied that the differences for the GY should be large enough for the analysis of MPH and BPH.

**Table 2:** Analysis of variance for grain yield for 95 crosses generated by crossing 5 testers (♂) with 19 lines (♀).

Source	DF	Mean Squares for GY†	
		GY	Pr(>F)
Locations (Loc)	2	924.90	6.08e-100
Replications: Loc	6	11.16	0.000339
Lines	18	186.74	5.68e-132
Testers (T)	4	33.48	6.20e-10
Lines × T	72	19.1	1.46e-45
Lines × Loc	36	9.65	2.56e-11
T × Loc	8	19.04	3.58e-09
Lines × T × Loc	144	8.55	2.52e-23
Error	564	2.63	

†GY, Grain yield (tons per ha).

### Selection of PEUS SNPs

**Figure 1:**
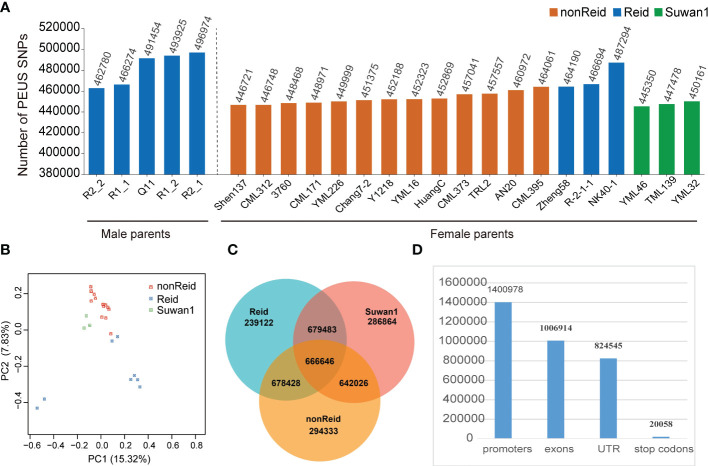
**(A)** Number of the PEUS SNPs in 5 male parent lines (Reid) and 19 female parent lines, which represent 3 heterotic groups (nonReid, Reid and Suwan1); **(B)** Principal component analysis (PCA) plot of 24 maize parent lines; **(C)** The mean number of heterozygous_PEUS_SNP in 3 heterotic groups (nonReid, Reid and Suwan1), and mean number of homozygous _PEUS_SNP among Suwan1, Reid and nonReid; **(D)** The total number of the PEUS SNPs in the promoters, exons, untranslated regions (UTRs), and stop codons regions.

To predict the MPH_GY and BPH_GY, we sequenced 24 maize parental lines from three different heterotic groups (**Figure 1A**) with a sequence depth of ~5×. A total of 58,986,791 high-confidence SNPs was retained after filtration (see Methods section). The numbers of the PEUS SNPs in the five testers and 19 lines are shown in **Figure 1A**. The results from **Figure 1A** showed that the number of the PEUS SNP were different in the five testers and among the 19 lines. This result was highly expected since the 24 inbreds belonged to three different heterotic groups (**Figure 1B**). The five testers and three lines from Reid heterotic group had more PEUS SNPs than those in the lines from both nonReid and Suwan1 heterotic groups (**Figure 1A**).

**Figure 2:**
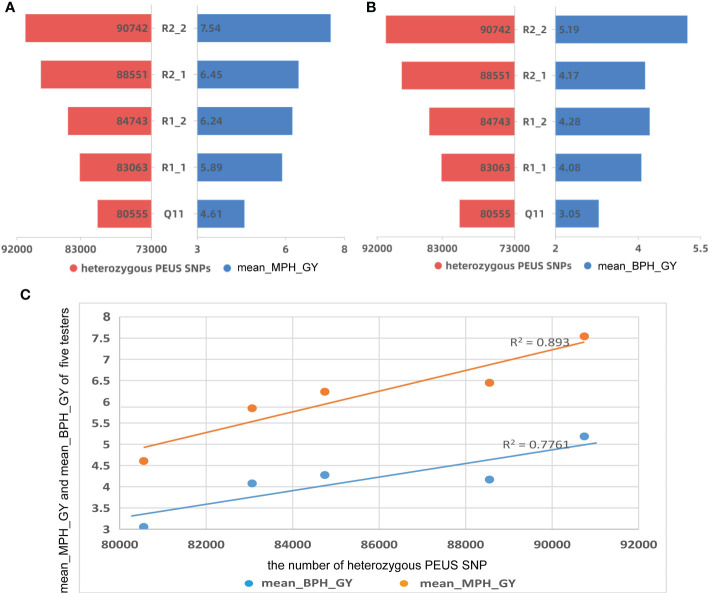
**(A)** The number of heterozygous PEUS SNP vs mean mid-parent heterosis of grain yield (MPH_GY); **(B)** The number of heterozygous PEUS SNP vs mean better-parent heterosis of grain yield (BPH_GY); **(C)** Regressions of MPH_GY and BPH_GY by the mean number of heterozygous PEUS SNPs.

**Figure 3:**
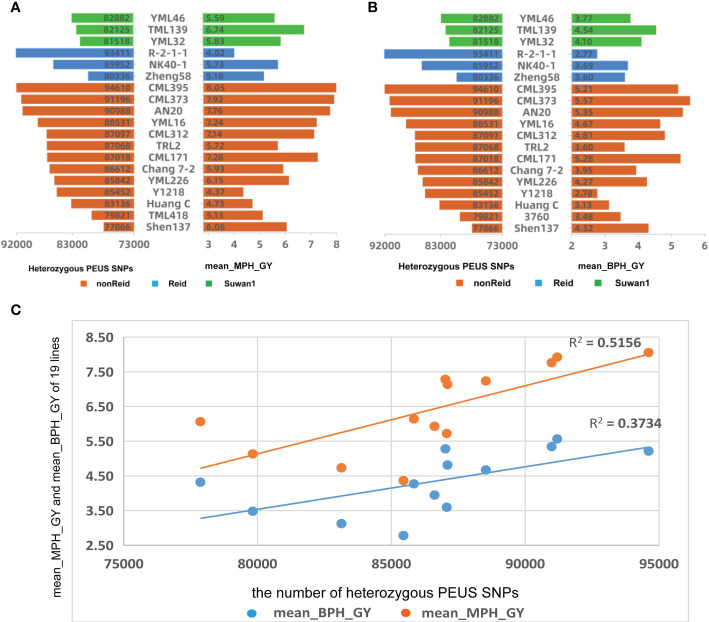
**(A)** Mean number of heterozygous PEUS SNP vs mean mid-parent heterosis of grain yield (MPH_GY); **(B)** Mean number of heterozygous PEUS SNP vs mean better-parent heterosis of grain yield (BPH_GY); **(C)** Regressions of mean MPH_GY and mean BPH_GY by the mean number of heterozygous PEUS SNPs of the crosses grouped by 13 lines from nonReid heterotic group.

The number of reads for each of the 24 inbred lines is summarized in **Supplemental Table 2**. The homozygous and heterozygous PEUS SNP are given in **Figure 1C**. Total numbers of reads from promoters, exons, untranslated regions (UTRs), and stop codons were accounted for (see **Figure 1D**). The results showed that the promoter had the highest number of PEUS SNP. This result matched with the findings of Li et al. (2022), who found that the promoters and the regions of 10–50 kb distance from the gene bodies had been changed and accumulated more favorable SNPs for maize inbred lines during the history of maize breeding. We notice that the promoter has the highest numbers of PEUS SNP (**Figure 1D**) that may lead to high MPH_GY and BPH_GY (**Figures 2**, **3**). Previous studies showed that 1) A multiple promoters may be involved to regulate the expression of one gene (Kühn et al., 2005); 2) A promoter may control the expression of several genes (Yang et al., 2008); 3) Individual exons in a same gene may be regulated by different promoters (Carol et al., 2000). The results from all these studies had suggested promoters are highly diversified. We can hypothesis that rich variations or polymorphisms are required by regulating different exon or different gene expressions in all creatures, especially in plant and human genomes.

Correlation between mean number of heterozygous PEUS SNPs or GD and mean MPH, BPH of GY from the 95 crosses averaged by the five testers and 19 lines at all locations

GY for all 95 crosses at each of the three locations, GD and mean GY across three locations are given in **Supplemental Table 1**. The mean number of heterozygous PEUS SNPs, mean MPH_GY, and mean BPH_GY for the five testers are depicted in **Figures 2A**, **B**. The results showed that 1) the four RILs had more heterozygous PEUS SNPs and higher mean MPH_GY and higher mean BPH_GY than their recurrent parent (Q11). These results suggested that introgression of genes from the tropical donor parent YML32 (Zhang et al., 2012) increased the diversity of genetic background of the original recurrent parental line Q11, which likely increased the mean MPH_GY and BPH_GY of the crosses involving the four RILs; 2) MPH_GY and BPH_GY seem to be related to the mean number of heterozygous PEUS SNPs of the five testers in crosses with the 19 lines. A statistical analysis further showed that MPH_GY and the mean number of heterozygous PEUS SNPs of the five testers were significantly correlated (r=0.942, p=0.017). Similar result was found between BPH_GY and heterozygous PEUS SNPs (r=0.881, p=0.048). A regression plot (**Figure 2C**) demonstrated high determination coefficients for both MPH_GY (0.89) and BPH_GY (0.78) when plotted against the mean number of heterozygous PEUS SNPs. These results suggested that the number of heterozygous PEUS SNPs could be used for predicting the mean MPH_GY and BPH_GY of hybrids from their inbred parental lines. With the rapid development of DNA sequencing technology and due to its cost-effectiveness, breeders can select potential inbreds with high heterozygous PEUS SNPs to make the crosses. Then there would be a better chance of developing hybrids with high capacity than using methods without PEUS SNP information. Thus, in practice, breeders may select good inbreds with PEUS SNPs before making actual crosses in the field, and this should help improve breeding efficiency to a great extent.

Similar analyses for the mean number of heterozygous PEUS SNPs, mean MPH_GY, and mean BPH_GY were conducted for the 95 crosses grouped by lines and three heterotic groups (**Figures 3A, B**). The Figure revealed that the high mean number of heterozygous PEUS SNPs in a line usually resulted in high mean MPH_GY and BPH_GY for a line in crosses with the testers, with a few exceptions. The trend was more obvious for the lines in the nonReid heterotic group than those in the other two heterotic groups. The difference might have happened since there were only three lines each in the Suwan1 and Reid heterotic groups. The correlations between the mean number of heterozygous PEUS_SNPs and MPH_GY and BPH_GY were calculated for the remaining 13 lines from the nonReid heterotic group. The results showed that the correlation between the mean number of heterozygous PEUS SNPs and MPH_GY was statistically significant (r=0.719, p=0.006). Similarly, the correlation between the mean number of heterozygous PEUS SNPs and BPH_GY was also found to be statistically significant (r=0.611, p=0.027). The regression plot for the mean MPH_GY and BPH_GY by the mean number of PEUS SNP for the 13 lines was constructed (**Figure 3C**). The determination coefficients for both mean MPH_GY (0.51) and BPH_GY (0.37) were smaller than those of the five testers. The possible reason for this is that the five testers were crossed with the 19 lines that belonged to three heterotic groups and were genetically highly diversified. On the contrary, the 13 lines had been crossed only with the lines from the five testers, and they all belonged to the Reid heterotic group and were less diverse. Thus, to effectively explore its potential of improving the GY of maize, the maize inbred with a higher number of heterozygous PEUS SNPs should be crossed with the lines with diverse genetic backgrounds or possibly from different heterotic groups.

### Correlation between number of heterozygous PEUS SNPs/GD and mean MPH, BPH of GY in 95 crosses

**Figure 4:**
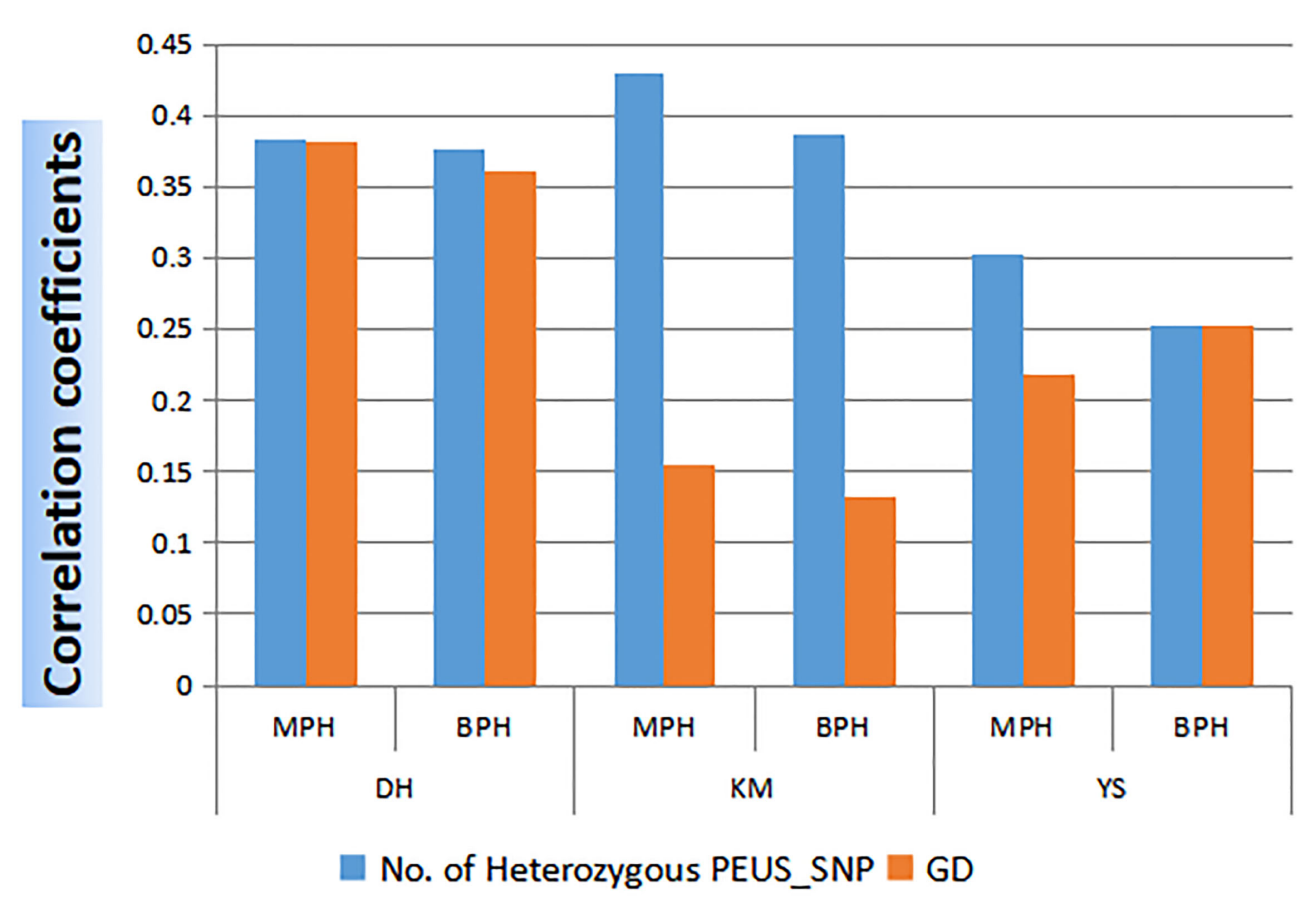
Correlation coefficients between the number of heterozygous PEUS SNP/GD and MPH/BPH of GY in 95 crosses at three locations.

Since GD is widely used for the prediction of heterosis, we calculated the correlation between GD and MPH_GY/BPH_GY in 95 crosses. Then we compared the correlation coefficient between the number of heterozygous PEUS SNP and MPH_GY/BPH_GY from the same 95 crosses at the three locations, Dehong (DH), Kunming (KM) and Yanshan (YS) (**Figure 4**). It showed that all the correlation coefficients were highly significant except GD at the KM location. The results revealed that the correlation coefficients were higher between the number of heterozygous PEUS_SNP and MPH_GY/BPH_GY than those between GD and MPH_GY/BPH_GY in all three individual locations. A t-test showed that the correlation coefficient of the number of heterozygous PEUS SNP was significantly higher than that of GD (P=0.03). The likely reason underlining the difference is that GD was calculated based on all genetic differences between two parents. In comparison, the number of heterozygous PEUS SNP was calculated from selected genomic regions between two parents (see Methods). Recently, Li et al. (2022) assembled 1,604 historically utilized maize inbred lines belonging to various female heterotic groups and male heterotic groups. They found that the frequency of favorable alleles at the associated SNPs exhibited convergent increases and accumulated in both the female and male heterotic groups for the convergently improved traits such as grain yield per plant (GYPP) and yield-related traits during modern maize breeding. Since the selected heterozygous PEUS SNPs are directly related to gene expressions (i.e., exons, promoters, stop codons), the chances of these SNPs likely covering most of the accumulated favorable alleles/genes are higher. As described by Li et al. (2022), this could have determined the GY of maize, whereas some other markers present in different genomic regions covered by GD were not included in the heterozygous PEUS SNPs in an inbred line. Therefore, our results strongly suggested that the number of heterozygous PEUS SNPs would be better for heterosis prediction than GD.

### Validation of the predictability of heterosis by the mean number of heterozygous PEUS SNP

We selected the top 10 crosses with high GY from our experiment and found that 7 out of the 10 crosses involved RIL R2_2 as the male parent, and 4 out of 10 crosses involved CML395 or CML 373 as the female parent. As expected, the RIL R2_2 had the highest mean number of heterozygous PEUS SNPs among the five testers (**Figure 2**), whereas CML395 and CML373 had the highest mean number of heterozygous PEUS SNPs among the 19 lines. These results showed that the maize breeder should be able to confidently select inbreds with an increased number of heterozygous PEUS SNPs to develop hybrids with the possibility of getting higher GY. With SNP technology being available worldwide, breeders can use high-throughput DNA sequencing technologies to obtain SNP information from each line and calculate the number of heterozygous PEUS SNPs for each inbred line. Then, the mean number of heterozygous PEUS SNP for all individual inbred lines could be computed and finally, the lines with a higher mean number of heterozygous PEUS SNP could be selected to make the crosses. By using PEUS SNP information, breeders would need to select fewer inbreds to make the required crosses to reach their breeding goals aimed at developing hybrids with higher GY. Thus, the breeding efficiency can be substantially improved. So, the results of the present investigation should have possible applications in maize breeding programs.

## Conclusion

In this study, we selected a set of SNPs in the promoters, exons, untranslated region, and stop codons (PEUS SNPs) regions and investigated whether the number of heterozygous PEUS SNP were correlated with mean MPH and mean BPH for GY, and were better in predicting the GY of maize compared to GD. The results showed that 1): the correlation coefficients between the number of PEUS SNPs and MPH_GY/BPH_GY were higher than those between GD and MPH_GY and BPH_GY in the 95 crosses at all three locations. Hence, this result implied that the number of PEUS_SNP would be a better predictor of heterosis than GD; 2) the mean number of heterozygous PEUS SNPs was highly correlated with mean MPH_GY and BPH_GY in the 95 crosses, either grouped by five testers (r = 0.942, p = 0.017<0.05) or grouped by 13 lines from the nonReid heterotic group (r = 0.612, p = 0.026<0.05). Within the 95 crosses, 7 hybrids out of the top 10 crosses with higher GY were found to be from three inbreds with the highest number of heterozygous PEUS SNPs. This result should be of great interest to the maize breeders; it should be useful for improving the breeding efficiency of maize by selecting fewer lines with a higher mean number of heterozygous PEUS SNP to obtain high-yielding crosses.

## Data availability statement

The datasets presented in this study can be found in online repositories. The names of the repository/repositories and accession number(s) can be found below: National Genomics Data Center, project ID PRJCA014289.

## Author contributions

FJ: Data collection and curation, software, writing- original draft preparation. XY: Data collection. ZL: Data collection. RG: Visualization. JW: Visualization. JF: Writing – review. YZ: Writing - review and editing. MK: Writing - review and editing. XF: Conceptualization, methodology. All authors contributed to the article and approved the submitted version.
